# Validity of Pneumonia Severity Assessment Scores in Africa and South Asia: A Systematic Review and Meta-Analysis

**DOI:** 10.3390/healthcare9091202

**Published:** 2021-09-11

**Authors:** Sarah Khalid Al Hussain, Amanj Kurdi, Nouf Abutheraa, Asma AlDawsari, Jacqueline Sneddon, Brian Godman, Ronald Andrew Seaton

**Affiliations:** 1 Strathclyde Institute of Pharmacy and Biomedical Sciences, University of Strathclyde, Glasgow G4 0RE, UK; Amanj.Baker@strath.ac.uk (A.K.); nouf.abutheraa@gmail.com (N.A.); Ph.d-afd@hotmail.com (A.A.); Brian.Godman@strath.ac.uk (B.G.); 2 Department of Pharmacy Practice, College of Clinical Pharmacy, King Faisal University, Hofuf 31982, Saudi Arabia; 3 Department of Pharmacology and Toxicology, College of Pharmacy, Hawler Medical University, Kurdistan Region Government, Erbil 44001, Iraq; 4 Division of Public Health Pharmacy and Management, School of Pharmacy, Sefako Makgatho Health Sciences University, Pretoria 0204, South Africa; 5 Security Forces Hospital Program, Riyadh 11481, Saudi Arabia; 6 AlKharj Maternity and Children Hospital, Ministry of Health, Riyadh 16278, Saudi Arabia; 7 Scottish Antimicrobial Prescribing Group, Healthcare Improvement Scotland, Delta House, 48 West Nile Street, Glasgow G1 2NP, UK; jacqueline.sneddon@nhs.scot (J.S.); Andrew.Seaton@ggc.scot.nhs.uk (R.A.S.); 8 School of Pharmaceutical Sciences, Universiti Sains Malaysia, George Town 11800, Malaysia; 9 Infectious Diseases Unit, Queen Elizabeth University Hospital, NHS Greater Glasgow & Clyde, 1345 Govan Road, Glasgow G51 4TF, UK; 10 Department of Medicine, University of Glasgow, Glasgow G12 8QQ, UK

**Keywords:** community-acquired pneumonia, severity of illness index, developing countries, mortality, prognosis, systematic review, meta-analysis

## Abstract

Background: Although community-acquired pneumonia (CAP) severity assessment scores are widely used, their validity in low- and middle-income countries (LMICs) is not well defined. We aimed to investigate the validity and performance of the existing scores among adults in LMICs (Africa and South Asia). Methods: Medline, Embase, Cochrane Central Register of Controlled Trials, Scopus and Web of Science were searched to 21 May 2020. Studies evaluating a pneumonia severity score/tool among adults in these countries were included. A bivariate random-effects meta-analysis was performed to examine the scores’ performance in predicting mortality. Results: Of 9900 records, 11 studies were eligible, covering 12 tools. Only CURB-65 (Confusion, Urea, Respiratory Rate, Blood Pressure, Age ≥ 65 years) and CRB-65 (Confusion, Respiratory Rate, Blood Pressure, Age ≥ 65 years) were included in the meta-analysis. Both scores were effective in predicting mortality risk. Performance characteristics (with 95% Confidence Interval (CI)) at high (CURB-65 ≥ 3, CRB-65 ≥ 3) and intermediate-risk (CURB-65 ≥ 2, CRB-65 ≥ 1) cut-offs were as follows: pooled sensitivity, for CURB-65, 0.70 (95% CI = 0.25–0.94) and 0.96 (95% CI = 0.49–1.00), and for CRB-65, 0.09 (95% CI = 0.01–0.48) and 0.93 (95% CI = 0.50–0.99); pooled specificity, for CURB-65, 0.90 (95% CI = 0.73–0.96) and 0.64 (95% CI = 0.45–0.79), and for CRB-65, 0.99 (95% CI = 0.95–1.00) and 0.43 (95% CI = 0.24–0.64). Conclusions: CURB-65 and CRB-65 appear to be valid for predicting mortality in LMICs. CRB-65 may be employed where urea levels are unavailable. There is a lack of robust evidence regarding other scores, including the Pneumonia Severity Index (PSI).

## 1. Introduction

Community-acquired pneumonia (CAP) is considered the leading cause of global deaths due to infectious diseases in all age groups, particularly in low- and middle-income countries (LMICs) [1]. Despite advances in pneumonia management and the development of a pneumococcal conjugate vaccine, pneumonia remains a major cause of adult hospitalisation and mortality worldwide [2]. According to the Global Burden of Diseases, Injuries, and Risk Factors Study 2016, more than 336 million episodes of lower respiratory tract infections (LRTIs) were reported globally, corresponding to 65.9 million hospitalisations and 2,377,697 deaths [3]. Reflecting the pneumococcal vaccination programme, death from LRTIs in children under five years of age has declined between 2007 and 2017 by more than 36%. Conversely, mortality in those aged 70 years and older has risen by 33.6% [4]. In sub-Saharan Africa, pneumonia accounts for approximately 4 million episodes and 200,000 deaths annually [2].

In high-income countries (HICs), the burden of CAP is high among the elderly, those with chronic obstructive pulmonary disease, and individuals with multiple comorbidities [5]. In contrast, indoor air pollution, crowding, malnutrition and high HIV prevalence, are considered the predominant risk factors in LMICs [6] and explain the higher disease burden amongst young and middle-aged adults in LMICs compared to HICs [2,7].

Several risk predictive scores/tools, such as Pneumonia Severity Index (PSI) and CURB-65, have been developed to facilitate site-of-care decision making, including predicting mortality, hospital admission need, and treatment intensity [8]. PSI [9], which consists of 20 variables including laboratory tests, places patients into five categories (I–V) for mortality, whereas CURB-65 [10] classifies patients into low-, intermediate- or high-risk groups based on five variables: confusion, urea, respiratory rate, blood pressure and age. Such scores support clinical judgement and aid the rationalisation of management decisions through patient risk categorisation [8]. This has been shown to improve the accuracy of triage to determine whether patients can be safely treated at home or require hospital admission, as well as support the appropriate selection of antimicrobial agents [11].

The use of severity assessment scores is of particular value in CAP management in LMICs, given its high prevalence coupled with *growing rates of antimicrobial resistance (AMR)* and limited or lack of access to laboratory, radiological diagnostics or advanced care settings such as intensive care units (ICU) [12]. Although widely used [6], the performance, validity and reliability of CAP scoring tools developed in HICs [8] are not well defined in LMICs. Such tools may be less suitable for use in LMICs since they have been derived from a HIC population with different population characteristics, such as age and ethnicity, comorbidity (including coinfection with HIV), nutritional status and tuberculosis prevalence/clinical overlap [13,14,15,16]. To date, we believe there has not been a comprehensive evaluation of the validity of CAP scoring tools in LMIC populations, despite some evidence showing their poor performance [8,17,18]. CRB-65 performed poorly in a Malawian hospital, where it was insensitive to predicting mortality compared to a locally developed score [19]. Furthermore, the inconsistent results arising from implementing these tools in LMICs, we believe, *support the need for a systematic evaluation of their validity in these specific populations* [2].

Herein, we systematically investigated the association between the various severity assessment scores and patient outcomes and subsequently evaluated their validity and predictive performance in adults with CAP in LMICs, particularly in Africa and South Asia. This will facilitate future guidance on their utility in LMICs and consideration of whether existing scoring tools need to be adapted for use in LMICs.

## 2. Materials and Methods

This systematic review and meta-analysis was performed in accordance with the PRISMA statement [20]. The protocol was registered with PROSPERO, CRD42020182620.

### 2.1. Search Strategy and Data Sources

Five electronic databases were systematically searched from inception up to 21 May, 2020. These included Medline (via Ovid), Embase (via Ovid), Cochrane Central Register of Controlled Trials, Scopus and Web of Science. Key terms and their synonyms were used for three concepts: CAP patients, severity assessment scores and low- and middle-income countries. The following combinations of search terms were used for Scopus: ((*“Community-acquired pneumonia”* OR “Bronchopneumoni*” OR “Pneumoni*” OR “Acute respiratory infection*” OR *“acute respiratory illness”* OR “lower respiratory tract infection*” OR “lower respiratory infection*”) AND (“low-middle-income countr*” OR “LMIC*” OR “low-income countr*” OR “less developed countr*” OR “middle-income countr*” OR *“Malawi”* OR *“Kenya”* OR *“Tanzania”* OR *“Africa”* OR *“South Africa”* OR “Developing countr*”) AND (“Prognos*” OR “Score*” OR “Tool*” OR *“severity assessment”* OR *“risk assessment”* OR “Predict*” OR “Mortality score*” OR “Severity score*” OR *“PSI”* OR *“CURB-65”* OR *“CURB65”* OR *“CRB65”* OR *“CRB-65”* OR *“SOAR”* OR *“SCAP”* OR *“PIRO”* OR *“RISC”* OR *“mRISC”* OR *“Pneumonia severity index”* OR *“I-DROP”*)). The search was limited to English language, with no additional restrictions. The search strategies were reviewed by two co-authors (NA, AK) and an expert academic librarian. The reference lists of relevant articles were screened in addition to supplementary, non-systematic hand-searching. The OpenGrey database was searched for unpublished literature. The full employed strategy is available in the Supplementary Materials.

### 2.2. Study Selection

#### 2.2.1. Eligibility Criteria

We included studies of any design (randomised control trials or observational studies) that involved adults with CAP and examined pneumonia severity scores performance to predict mortality, hospitalisation, ICU admission, mechanical ventilation or treatment intensity. Additionally, the included studies were undertaken in LMICs, in Africa or South Asia, as they represent the majority of the countries in the LMICs list by 46% and 12%, respectively, according to the World Bank classification [21]. These countries also account for the highest mortality secondary to LRTIs, including pneumonia [3]; there, it is crucial to improve the appropriate use of antimicrobials due to rising rates of antimicrobial resistance [22]. Qualitative studies, abstracts, reports, commentaries, editorials and book chapters were excluded. We also excluded studies that included patients with other types of pneumonia, such as hospital-acquired, healthcare-associated, ventilator-associated or aspiration pneumonia, or if a single prognostic factor or other biomarkers were used instead of the clinical scores.

#### 2.2.2. Screening

All identified records were imported into Covidence^®^ (www.covidence.org), accessed on 25 May 2020, where duplicate citations were removed. Titles and abstracts, followed by full-text screenings, were performed by the principal author (SA). Co-authors (NA, AA) independently validated the selection by screening a randomly selected sample of 20% at each stage. 

### 2.3. Data Extraction and Quality Assessment

Data were extracted into Excel spreadsheets by the principal author (SA), including study characteristics (first author, year, country, study design, setting, population characteristics and sample size), severity score, CAP definition, study outcomes, including mortality, ICU admission, hospitalisation, treatment intensity, mechanical ventilation need and time to clinical stability and, if possible, true positive (TP), false positive (FP), false negative (FN), and true negative (TN) values. These values were tabulated for patients with high-risk (CURB-65 ≥ 3 and CRB-65 ≥ 3) and intermediate-risk (CURB-65 ≥ 2 and CRB-65 ≥ 1) cut-offs. Methodological quality of the studies was assessed using Quality in Prognosis Studies (QUIPS) criteria [23], a tool recommended by the Cochrane Prognosis Methods Group [24]. This tool consists of six domains, where each has a score from 0 to 2. As used by Marti et al. [25], studies with an overall score between 11 and 12, 9 and 10, or 8 or less were considered of low-, moderate-, or high-risk of bias, respectively. Independently, co-authors (NA, AA) validated the extraction and quality assessment of a 20% randomly selected sample. For any disagreement, author (AK) was involved until consensus was achieved.

### 2.4. Data Analysis

When at least four studies (a minimum number required to use MIDAS [26] command) were available for each scoring tool and outcome, the performance of the identified tools was assessed in two ways: firstly, the association between different severity scores at the studied cut-offs and the reported event (mortality) was examined using pooled relative risks (RRs). Furthermore, a bivariate model was used to calculate the scores’ performance characteristics, including the pooled sensitivity, specificity, positive likelihood ratios (PLRs), negative likelihood ratios (NLRs) and diagnostic odds ratios (DORs). Area under the receiver operating characteristic (AUROC) curve was obtained to evaluate the overall scores’ accuracy. The results were described as point estimates and 95% confidence intervals. Heterogeneity was tested using I^2^ index, where a value of <25%, 25–50%, and >50% indicated low, moderate, and high heterogeneity, respectively [27]. Data were combined using the random-effects model when I^2^ > 50%. When meta-analysis could not be conducted due to the nature of the available data or the small number of studies, the results were narratively summarised. Publication bias was explored using Deeks’ funnel plot [28], where a *p*-value < 0.05 indicated the presence of bias. All analyses were carried out in STATA IC 16.1 (Stata Corp, College Station, TX, USA), where the MIDAS [26], which can be applied only to data from a minimum of four studies, and metan commands were used. 

## 3. Results

### 3.1. Search Results

**Figure 1:**
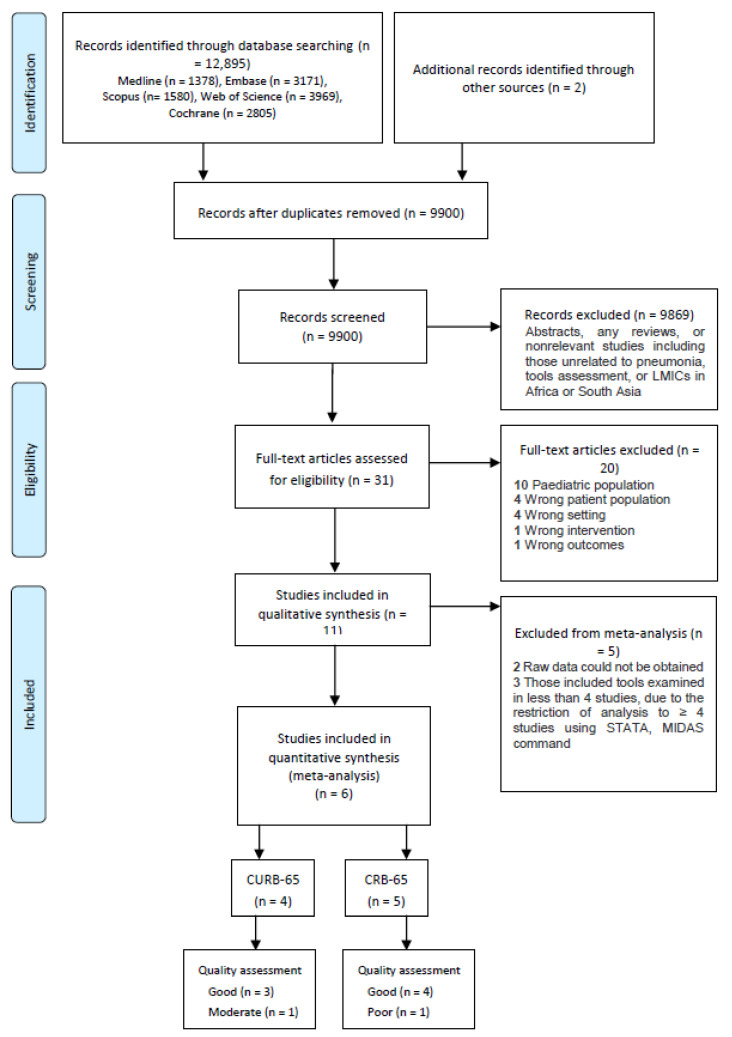
PRISMA flow diagram for the study selection process.

Titles and abstracts of 9900 records were screened against the inclusion criteria after deduplication; however, only 31 studies were considered for full-text screening. Of these, 11 studies fulfilled the eligibility criteria; however, only 6 studies that examined CURB-65 and CRB-65 included sufficient data and were included in the final meta-analysis [19,29,30,31,32,33]. The study selection is summarised in Figure 1.

### 3.2. Study Characteristics

The eligible 11 studies were published between 2008 and 2019, with a total of 3740 patients from 7 LMICs. Eight studies were conducted in Africa (Malawi [18,19,34], Nigeria [30], South Africa [29,33], Uganda [17] and Egypt [35]), and three were from South Asia (Pakistan [32] and India [31,36]). The average age of the patients ranged from 34 to 69.9 years, and male percentage varied between 38.6% and 62.1%. The reported mortality rate ranged from 2–40%. Most of identified studies assessed patients in medical wards, emergency departments or outpatient settings. Only one study exclusively evaluated elderly patients (≥60 years) admitted to ICU [35]. A total of 12 scores, CURB-65 [30,31,32,33,34,35], CRB-65 [19,29,30,32,33,34], PSI [31,36], SWAT-Bp [18,19,34], CURB-45 [33], SCAP [35], ADL score [35], modified IDSA/ATS criteria [34], Koss et al. tool [17], CTA [33], ACHU [33] and SMRT-CO [34], were examined in these 11 studies, 7 of which reviewed the performance of more than one score [19,30,31,32,33,34,35]. All studies addressed mortality as either in-hospital [18,19,33], 30-day [17,30,32,34,35], in-hospital or within 30 days of discharge [31,36] or in-hospital or within 14 days following emergency department visit for those discharged earlier [29]. Four studies included other outcomes (ICU admission [30,31], mechanical ventilation [35], hospitalisation and time to clinical stability [29]). Table 1 summarises the studies characteristics (additional characteristics in the Supplementary Materials (Table S1)). 

### 3.3. Methodological Quality

Studies of any quality were included in the meta-analysis. Risk of bias was considered low in five studies (score ≥ 11), moderate in four studies (score 9–10), and high in two studies (score ≤ 8). Quality assessment is described in the Supplementary Materials (Table S2).

### 3.4. Study Outcome

Although 12 severity scores were initially identified (scores’ components are provided in the Supplementary Materials Table S3), only two of them (CURB-65, CRB-65) were examined in four studies or more. In addition, only a few studies assessed outcomes other than mortality. Such scores and outcomes were excluded from the meta-analysis, with their findings reported narratively in the Supplementary Materials (Table S4). Consequently, out of the scores identified, the meta-analysis was only performed on CURB-65 and CRB-65 in predicting mortality.

### 3.5. Analysis of the Outcome

#### 3.5.1. Association between CURB-65/CRB-65 and Mortality

**Figure 2:**
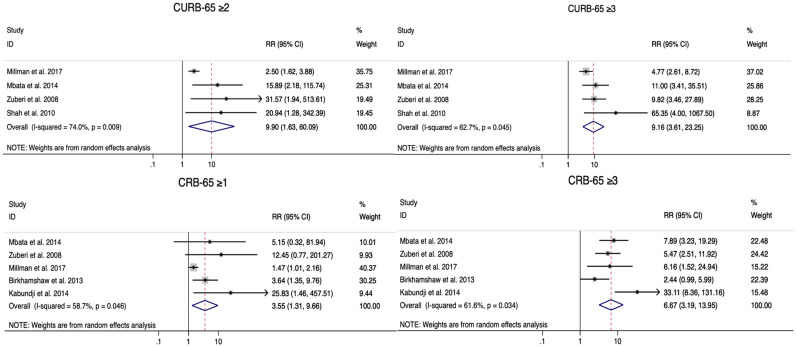
Forest plots of the association between CURB-65 and CRB-65 at the studied cut-offs and mortality prediction in patients with community-acquired pneumonia.

All studies included in the meta-analysis (four for CURB-65 and five for CRB-65) showed that the high-risk class (CURB-65 ≥ 3, CRB-65 ≥ 3) was associated with increased mortality, with pooled RRs of 9.16 (3.61–23.25) and 6.67 (3.19–13.95) for CURB-65 and CRB-65, respectively. The intermediate-risk class (CURB-65 ≥ 2, CRB-65 ≥ 1) was also related to high mortality risk, with pooled RRs of 9.90 (1.63–60.09) and 3.55 (1.31–9.66) for CURB-65 and CRB-65, respectively. Due to the significant heterogeneity, the random-effects model was used (Figure 2).

#### 3.5.2. CURB-65 Predictive Performance for Mortality

**Figure 3:**
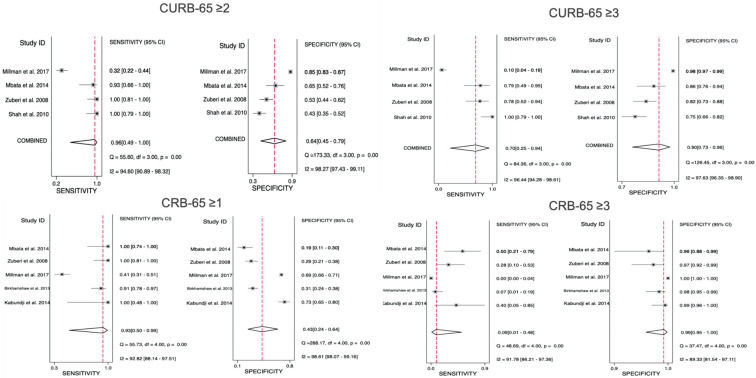
Forest plots for the sensitivity and specificity of CURB-65 and CRB-65 at the studied cut-offs for mortality prediction.

**Figure 4:**
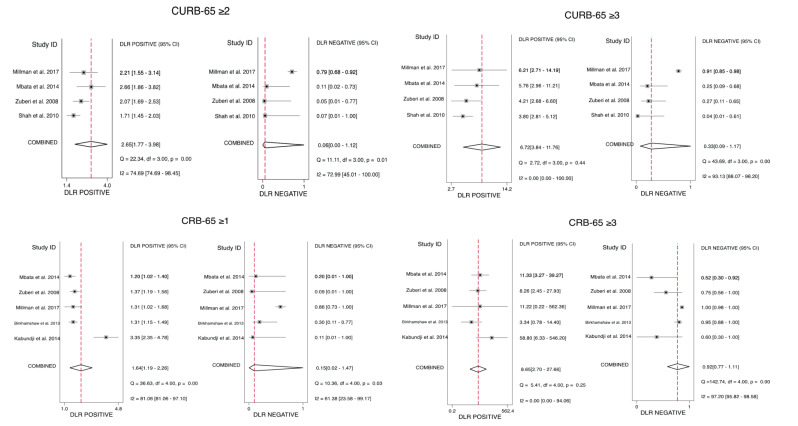
Forest plots for the positive and negative likelihood ratio of CURB-65 and CRB-65 at the studied cut-offs for mortality prediction.

**Figure 5:**
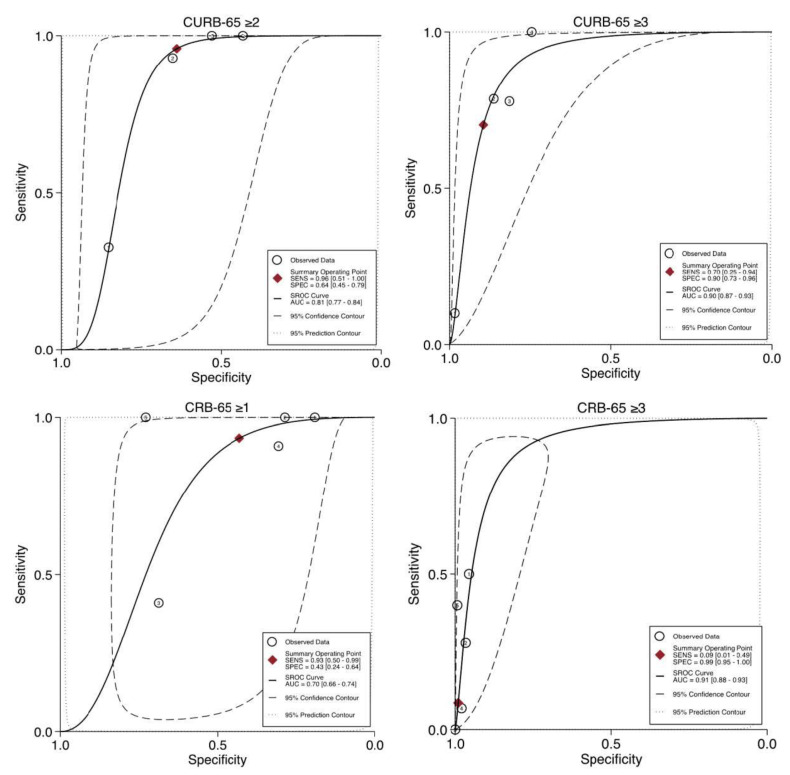
Area under the receiver operating characteristic (AUROC) curves for the included studies examining CURB-65 and CRB-65 at the studied cut-offs for mortality prediction. The numbers in the circle refer to the included studies.

From the eligible 11 studies, CURB-65 was assessed in 6 studies; however, 2 were excluded due to lack of data necessary to obtain the performance characteristics. Only four studies were finally analysed, with a total of 1378 patients. Two of these studies excluded HIV patients. The score performance characteristics are presented in Table 2. High-risk cut-off (≥3) showed better specificity, PLR, and AUROC of 0.90 (95% CI 0.73–0.96), 6.72 (95% CI 3.84–11.76) and 0.90 (95% CI 0.87–0.93), respectively. On the other hand, intermediate-risk cut-off (≥2) had an improved sensitivity and NLR of 0.96 (95% CI 0.49–1.00) and 0.06 (95% CI 0.00–1.12), respectively. Forest plots of the performance characteristics and the receiver operating characteristic curves are presented in Figure 3, Figure 4 and Figure 5 and the Supplementary Materials Figure S1.

#### 3.5.3. CRB-65 Predictive Performance for Mortality

Similarly, of the 11 studies, 6 studies examined CRB-65 performance. However, only five studies included sufficient data on performance and were eligible for analysis, involving a total of 1941 patients. HIV patients were excluded from one of the analysed studies. Similar to CURB-65, CRB-65 high-risk cut-off (≥3) showed higher specificity, PLR, and AUROC of 0.99 (95% CI 0.95–1.00), 8.65 (95% CI 2.70–27.66), and 0.91 (95% CI 0.88–0.93), respectively. In contrast, higher sensitivity and better NLR of 0.93 (95% CI 0.50–0.99) and 0.15 (95% CI 0.02–1.47), respectively, were seen with the intermediate-risk cut-off (≥1). Pooled performance characteristics for each studied cut-off are summarised in Table 2. Forest plots and the receiver operating characteristic curves are available in Figure 3, Figure 4 and Figure 5 and the Supplementary Materials Figure S1.

### 3.6. Publication Bias

The presence of publication bias was assessed by Deeks’ funnel plot (the Supplementary Materials Figure S2). The funnel plots for CURB-65 and CRB-65 at high-risk cut-offs did not show any evidence of bias (*p* = 0.18 and 0.48, respectively). However, the plots’ shape at their intermediate-risk cut-offs revealed asymmetry (*p* = 0.04 and 0.03, respectively).

## 4. Discussion

To the best of our knowledge, this is the first systematic review and meta-analysis to summarise the existing evidence regarding the validity and performance of available pneumonia severity scoring tools in LMICs. The analysis demonstrates that CURB-65 and the simplified CRB-65 at their high- and intermediate-risk cut-offs are useful to predict higher mortality risk, with a stronger association observed with CURB-65. These findings suggest that both scores can be used to identify patients at increased risk of mortality in LMICs to help guide their future management. This builds on the findings predominately from HICs. Chalmers et al. did not reveal meaningful differences following the evaluation of 30-day mortality prediction performance of PSI, CURB-65 and CRB-65 based on an analysis of 40 studies [8]. Similarly, Loke et al. explored different severity scores’ performance, including CURB-65 and CRB-65, in predicting mortality by analysing 23 studies and produced a similar conclusion [37]. Both meta-analyses [8,37], though, included only a single LMIC study conducted in Pakistan [32], which was also included in our meta-analysis.

According to our AUROC findings, intermediate- and high-risk scores’ cut-offs displayed excellent accuracy for CURB-65 (0.81 and 0.90, respectively) and acceptable to outstanding accuracy for CRB-65 (0.70 to 0.91, respectively) in predicting mortality among patients with CAP [38]. At high-risk cut-off (≥3), no substantial difference was observed between the scores’ performance, with AUROC greater than those reported by Ebell et al., who examined the discrimination of CRB-65 by analysing 29 studies, excluding studies from low-income and lower-middle-income countries, and Chalmers et al., whereas CRB-65 at intermediate-risk cut-off (≥1) had the lowest AUROC [8,39]. These differences may be attributed to the variations in population characteristics, particularly patient age and comorbidities.

Our analysis also revealed differences in the performance characteristics among the assessed scores (CURB-65, CRB-65). Both scores appear to have improved specificity at their high-risk cut-offs (CURB ≥ 3, CRB-65 ≥ 3), suggesting that they correctly identify patients who are not at increased risk of mortality. However, the relatively poor sensitivity, particularly for CRB-65, may lead to misclassifications and poor management of possibly high-risk patients, which may limit their utility in clinical practice and decision making in LMICs. Contrastingly, better sensitivity and lower specificity are seen at their intermediate-risk cut-offs (CURB-65 ≥ 2, CRB-65 ≥ 1). In terms of likelihood ratios, CURB-65 and CRB-65 showed better PLRs at their high-risk cut-offs, with superiority for the latter (6.72 vs. 8.65), suggesting that CRB-65 performs better in this aspect, although a PLR of greater than 10 is essential [40]. Both scores at the studied cut-offs yielded NLRs of less than one; however, based on previous findings, only CURB-65 (≥2) had a robust result of less than 0.1 [40].

According to our results, it seems likely that both scoring systems could be used in LMICs for mortality prediction, as they both support appropriate management approaches. Overall, high-risk cut-offs are useful to allocate high-mortality-risk patients to a higher level of care unit, such as high-dependency units in HICs, where beds are available. However, in facilities where such units and resources are not accessible, these cut-offs may be employed to support other management decisions such as intravenous administration of antibiotics, if available. Intermediate-risk cut-offs might be a more practical and valuable option to guide hospitalisation for patients in LMICs, as this would reduce the risk of increased mortality among individuals with CAP.

Investigating other scores performance and outcomes in LMICs was not possible. The well-known and validated PSI tool has not yet been extensively studied in LMICs. PSI implementation also requires multiple clinical and laboratory variables [9], which are typically impractical to obtain in resource-scarce areas, especially if patients are charged for tests. Newer assessment tools, such as SWAT-Bp and SCAP, have been assessed in a very few studies in LMICs, and some have shown good discrimination ability. However, despite these promising results, the lack of evidence evaluating these scores limits their generalisability, and further studies are required to validate and establish their role among such populations. Marti and colleagues assessed several severity scores to predict early mortality (<14 days), ICU admission and treatment intensity, and found that newer scores, such as ATS/IDSA 2007 minor criteria, SCAP and SMART-COP, performed better compared to the classical tools (PSI, CURB-65) [25]. However, again, all but one study was conducted in HICs, and the majority addressed ICU admission as their outcome.

Our findings identified gaps in the existing literature warranting future research. The main issue is the small number of studies evaluating severity scores in LMICs. As a result, it was not possible to study most of the identified scores’ validity, particularly the newly developed and refined ones. It was also impractical to examine their use in predicting other important outcomes as we initially planned in our published study protocol (PROSPERO protocol, CRD42020182620), such as ICU admission, hospitalisation and treatment intensity, since all of the eligible studies only evaluated mortality as the main outcome. This was disappointing; however, this itself highlights the gap in the available evidence for using these tools for their intended purpose and emphasises the need for future research to see how these tools can be utilised to assess other outcomes. Due to the limited resources available in LMICs, attempts to develop strategies to improve CRB-65 (≥3) sensitivity, which does not require any laboratory results, are encouraged. Of note, our analyses were limited to adult populations; the performance of these prognostic scoring systems in paediatric patients in LMICs was not assessed in this report.

We are aware that our study has some potential limitations. We initially excluded a large number of studies, which could be explained by the fact that the search terms were wide and searched both as subject headings and free-text terms to ensure a complete and comprehensive search strategy. Another significant limitation is the *substantial heterogeneity* amongst the studies, which may have affected the results. However, high levels of heterogeneity are often seen in diagnostic test accuracy reviews [41]. In addition to the considerable variability in the patients’ eligibility criteria, study designs and settings, differences were found in mortality definition and pneumonia diagnostic criteria between studies. This is possibly due to the limited resources available in such settings as well as the lack of reliable and timely patients’ records, which in turn could result in the inclusion of misdiagnosed patients and patients with alternative LRTIs. Furthermore, HIV-infected patients were excluded from several studies included in our systematic review and meta-analysis, which may not represent the actual population demographics, considering the high prevalence of HIV especially in sub-Saharan African countries. Additional analyses stratified based on these differences were not possible to conduct due to the limited studies identified, which may have affected the findings of our work. Moreover, most of the included studies failed to provide details about management approaches, such as antibiotic treatment regimens and any oxygen, fluids, electrolytes or cardiovascular support needed, which may have influenced patient outcomes. Lastly, although changing the cut-off point from four to three analysed studies was unlikely to affect our results since none of the other scores were examined in three eligible studies, changing the cut-off to two studies would have allowed us to evaluate additional scores, namely, PSI and SWAT-Bp, as they were examined in two studies. However, obtaining pooled estimates for performance characteristics (sensitivity, specificity) requires at least four studies as recommended by MIDAS command in STATA [26].

## 5. Conclusions

Despite the differences in the scores’ performance characteristics, we found that CURB-65 and CRB-65 appear to be valid prognostic scoring systems for predicting death among adults with CAP in LMICs. Although CURB-65 exhibited a stronger association with mortality prediction and better performance in many aspects, this review suggests that the simple and readily available CRB-65 is also an appropriate score to employ where limited access to laboratory tests means that urea levels are unavailable. Given the differences in population characteristics and the limited resources available, further research is needed to address other important outcomes and to develop, adjust and validate other scores that are easier to use in such settings. We will be following such developments in the future.

## Figures and Tables

**Table 1:** Characteristics of the included studies.

Author. (Year)	Country	Study Settings	Study Design	Age in Years	Male n (%)	Sample Size	Assessed Score(s)	Outcome(s)	Mortality Definition	Mortality Rate (%)
Abd-El-Gawad (2013) [35]	Egypt	Ain Shams University Hospitals	Prospective cohort	69.9 (±11.4)	42 (60)	65	CURB-65, SCAP and ADL	Mortality and MV	30-day mortality	40
Aston (2019) [34]	Malawi	Queen Elizabeth Central Hospital	Prospective observational	34.7 (29.4–41.9) ^a^	285 (62.1)	459	CURB-65, CRB-65, SMRT-CO, SWAT-Bp and Modified IDSA/ATS	Mortality	30-day mortality	14.6 ^b^
Birkhamshaw (2013) [19]	Malawi	Medical admission ward of Queen Elizabeth Central Hospital	Retrospective	37 (29–48) ^a^	116 (48.3)	240	SWAT-Bp and CRB-65	Mortality	In-hospital mortality	18.3
Buss (2018) [18]	Malawi	Medical admission ward of Queen Elizabeth Central Hospital	Prospective cohort	35 (16–79)	90 (41.7)	216	SWAT-Bp	Mortality	In-hospital mortality	12.5
Kabundji (2014) [29]	South Africa	ED at Helen Joseph Hospital	Prospective observational	36.5 (20–87)	73 (48.0)	152	CRB-65	Mortality, hospital admission and time to clinical stability	During hospitalisation or 2 weeks after ED visit	3.3
Koss (2015) [17]	Uganda	Mulago Hospital	Prospective cohort	Mean: 34	389 (46.6)	835	Koss et al. new score	Mortality	30-day mortality	18.2
Mbata (2014) [30]	Nigeria	The Accident and Emergency, medical outpatients and medical wards of the University of Nigeria Teaching Hospital	Prospective observational	56 (±18)	39 (48.8)	80	CURB-65 and CRB-65	Mortality and ICU admission	30-day mortality	15
Millman (2017) [33]	South Africa	Tshepong Hospital, Chris Hani Baragwanath Academic Hospital, and Selby Hospital	Retrospective chart review	NR	2780 (38.6)	1356	CURB-65, CRB-65, CTA, CURB-45 and ACHU	Mortality	In-hospital mortality	7.4
Rajarajan (2017) [36]	India	A tertiary care hospital	Prospective observational	43.38 ± 16.43	29 (58)	50	PSI	Mortality	In-hospital or within 30 days of discharge	2
Shah (2010) [31]	India	Out- and in-patient departments of Sher-i-Kashmir Institute of Medical Sciences	Prospective study	60.8 (±13.6)	89 (59.3)	150	CURB-65 and PSI	Mortality and ICU admission	In-hospital or within 30 days of discharge	10.7
Zuberi (2008) [32]	Pakistan	Aga Khan University Hospital,	Longitudinal observational cohort	60.4 (±18.5)	65 (47.7)	137	CURB-65 and CRB-65	Mortality	30-day mortality	13.1

Age data are expressed in either median (range/interquartile range (IQR)) or mean ± standard deviation (SD); NR: Not reported; n: number of patients; MV: mechanical ventilation; ICU: intensive care unit; ED: emergency department; IQR: interquartile range; SCAP: severe community-acquired pneumonia; ADL: activities of daily living score; CURB-65: confusion, urea, respiratory rate, blood pressure, age ≥ 65; CRB-65: confusion, respiratory rate, blood pressure, and age ≥ 65; SMRT-CO: systolic blood pressure, multilobe infiltrate, respiratory rate, tachycardia, confusion, oxygen; SWAT-Bp: sex, muscle wasting, non-ambulatory, temperature, and blood pressure; IDSA/ATS: Infectious Diseases Society of America/American Thoracic Society; CTA: classification tree analysis; ACHU: Age, Confusion, HIV, Urea; PSI: Pneumonia Severity Index. ^a^ IQR; ^b^ only 439 patients were assessed for 30-day mortality.

**Table 2:** Pooled performance characteristics of CURB-65 and CRB-65 for predicting mortality in high- and intermediate-risk cut-offs in community-acquired pneumonia patients.

	High-Risk Cut-Offs		Intermediate-Risk Cut-Offs	
	CURB-65 ≥ 3	CRB-65 ≥ 3	CURB-65 ≥ 2	CRB-65 ≥ 1
Pooled Estimate	Summary Statistic	Summary Statistic	Summary Statistic	Summary Statistic
Sensitivity (95% CI)	0.70 (0.25–0.94)	0.09 (0.01–0.48)	0.96 (0.49–1.00)	0.93 (0.50–0.99)
Specificity (95% CI)	0.90 (0.73–0.96)	0.99 (0.95–1.00)	0.64 (0.45–0.79)	0.43 (0.24–0.64)
PLR (95% CI)	6.72 (3.84–11.76)	8.65 (2.70–27.66)	2.65 (1.77–3.98)	1.64 (1.19–2.26)
NLR (95% CI)	0.33 (0.09–1.17)	0.92 (0.77–1.11)	0.06 (0.00–1.12)	0.15 (0.02–1.47)
DOR (95% CI)	20.19 (7.32–55.63)	9.36 (2.57–34.03)	41.02 (2.87–586.97)	10.70 (1.04–109.87)
AUROC (95% CI)	0.90 (0.87–0.93)	0.91 (0.88–0.93)	0.81 (0.77–0.84)	0.70 (0.66–0.74)

CI: Confidence interval; PLR: positive likelihood ratio; NLR: negative likelihood ratio; DOR: diagnostic odds ratio; AUROC: area under the receiver operating characteristic curve; CURB-65, confusion, urea, respiratory rate, blood pressure, age ≥ 65 years; CRB-65, confusion, respiratory rate, blood pressure, age ≥ 65 years.

## Data Availability

Not applicable.

## Supplementary Materials

The following are available online at https://www.mdpi.com/article/10.3390/healthcare9091202/s1, Search Strategy: Search terms and Results, Table S1: Additional study characteristics, Table S2: Quality assessment of the included studies, Table S3: Components of the identified scoring systems, Table S4: Summary of the extracted results, Review of other scores’ performance, Figure S1: Forest plots of the diagnostic odds ratios (DORs), Figure S2: Deek’s funnel plots–Publication bias assessment.
