# Supplementary material for: Validity of Pneumonia Severity Assessment Scores in Africa and South Asia: A Systematic Review and Meta-Analysis
Source: Healthcare (Basel). 2021 Sep 11;9(9):1202. doi: 10.3390/healthcare9091202 (PMC8467534; doi:10.3390/healthcare9091202)
Supplement: Supplementary file 1 [file healthcare-09-01202-s001.zip › healthcare-1336868-SI.pdf]

Supplementary material

Search Strategy: Search Terms and Results – closing search date May 21, 2020

Medline Ovid: May 21, 2020

#	Search terms	Results
1	Pneumonia/	47,101
2	Community-Acquired Infections/	14,068
3	Respiratory Tract Infections/	38,202
4	Risk Assessment/	261,614
5	"Severity of Illness Index"/	240,279
6	Prognosis/	502,687
7	Decision Making/	94,189
8	Decision Trees/	11,052
9	Developing Countries/	74,354
10	Poverty Areas/ or Poverty/	42,851
11	"Africa South of the Sahara"/ or Africa/	36,982
12	Asia/	28,029
13	(Pneumonia OR Community-acquired pneumonia OR CAP OR Community-acquired infection* OR Respiratory tract disease* OR Respiratory infection*).ti,ab.	173,425
14	(Severity score* OR Predict* OR Prognosis OR Mortality score* OR CURB65 OR CURB 65 OR CURB-65 OR C-U-R-B-65 OR CRB65 OR CRB 65 OR CRB-65 OR C-R-B-65 OR Pneumonia severity index OR PSI OR PORT score OR Fine Score OR Tool* OR decision tree OR SOAR OR A-DROP OR PIRO OR RISC OR mRISC OR APPIS OR SCAP OR SMART-COP OR SWAT-Bp OR REA-ICU OR clinical prediction rule* OR clinical prediction score* OR clinical prediction tool* OR clinical decision rule* OR clinical decision score* OR severity assessment score* OR model* OR risk stratification* OR Prognostic scoring system* OR Severity prognosis OR Risk score* OR Severity index OR Prognostic tool* OR Severity of illness index).ti,ab.	4,801,450
15	(Developing OR less* developed OR under developed OR underdeveloped OR underserved OR under served OR deprived OR poor* OR least economically developed OR nonindustriali?ed OR non-industriali?ed OR middle income OR low* income OR lower middle-income OR low-middle-income OR Low middle income OR global south OR third world OR resource* poor OR resource* limited OR scarce* resource* OR low resourced OR LAMI) adj (countr* OR nation* OR population* OR world OR setting*).ti,ab.	109,854
16	(LAMIC* OR LMIC* OR Poverty area* OR Malawi OR Uganda OR Kenya OR Sub-Saharan OR Africa OR Asia).ti,ab.	189,997
17	(low adj3 middle adj3 countr*).ti,ab.	16,145
18	(low* adj (gdp or gnp or gross domestic or gross national)).ti,ab.	241
19	Transitional countr*.ti,ab.	159
20	1 OR 2 OR 3 OR 13	226,038
21	4 OR 5 OR 6 OR 7 OR 8 OR 14	5,403,763
22	9 OR 10 OR 11 OR 12 OR 15 OR 16 OR 17 OR 18 OR 19	375,479
23	20 AND 21 AND 22	1,393
24	Limit 23 to English language	1,378

Web of Science All Database: May 21, 2020

#	Search terms	Results
1	TS=("Pneumoni*" OR "Bronchopneumoni*" OR "Community-acquired pneumonia" OR "Acute respiratory infection*" OR "lower respiratory tract infection*" OR "lower respiratory infection*" OR "acute respiratory illness")	431,973
2	TS=(“Developing countr*" OR "LMIC*" OR "low-middle-income countr*" OR "Malawi" OR "Kenya" OR "Tanzania" OR "South Africa" OR "Africa" OR "low-income countr*" OR "less developed countr*" OR "middle-income countr*")	2,132,370
3	TS=("severity assessment" OR "risk assessment" OR "Prognos*" OR "Score*" OR "Tool*" OR "Predict*" OR "Pneumonia severity index" OR "PSI" OR "CURB65" OR "CURB-65" OR "CRB65" OR "CRB-65" OR "SOAR" OR "I-DROP" OR "SCAP" OR "PIRO" OR "RISC" OR "mRISC" OR "Mortality score*" OR "Severity score*")	10,201,396
4	#1 AND #2 AND #3	3,920

Scopus: May 21, 2020

#	Search terms	Results
1	TITLE-ABS-KEY ((*"Pneumoni*"* OR *"Bronchopneumoni*"* OR *"Community-acquired pneumonia"* OR *"Acute respiratory infection*"* OR *"lower respiratory tract infection*"* OR *"lower respiratory infection*"* OR *"acute respiratory illness"*) AND (*"Developing countr*"* OR *"LMIC*"* OR *"low-middle-income countr*"* OR *"Malawi"* OR *"Kenya"* OR *"Tanzania"* OR *"South Africa"* OR *"Africa"* OR *"low-income countr*"* OR *"less developed countr*"* OR *"middle-income countr*"*) AND (*"severity assessment"* OR *"risk assessment"* OR *"Prognos*"* OR *"Score*"* OR *"Tool*"* OR *"Predict*"* OR *"Pneumonia severity index"* OR *"PSI"* OR *"CURB65"* OR *"CURB-65"* OR *"CRB65"* OR *"CRB-65"* OR *"SOAR"* OR *"I-DROP"* OR *"SCAP"* OR *"PIRO"* OR *"RISC"* OR *"mRISC"* OR *"Mortality score*"* OR *"Severity score*"*))	1,614
2	Limit 1 to English language	1,580

Embase Ovid: May 21, 2020

#	Search terms	Results
1	Pneumonia/ or community acquired pneumonia/ or infectious pneumonia/	173,090
2	acute respiratory tract disease/	2,035
3	respiratory tract disease/ or lower respiratory tract infection/	66,409
4	PROGNOSIS/	568,232
5	risk assessment/	555,723
6	disease classification/ or "severity of illness index"/ or disease severity/ or scoring system/	892,998
7	decision making/	221,591
8	"decision tree"/	12,618
9	developing country/	89,383
10	"Africa south of the Sahara"/ or Africa/	62,744
11	Poverty Areas/ or Poverty/	44,220
12	Asia/	68,893
13	(Pneumonia OR Community-acquired pneumonia OR CAP OR Community-acquired infection$ OR Respiratory tract disease$ OR Respiratory infection$).ti,ab.	237,476
14	(Severity score$ or Predict$ or Prognosis or Mortality score$ or CURB65 or CURB 65 or CURB-65 or C-U-R-B-65 or CRB65 or CRB 65 or CRB-65 or C-R-B-65 or Pneumonia severity index or PSI or PORT score or Fine Score or Tool$ or decision tree or SOAR or A-DROP or PIRO or RISC or mRISC or APPIS or SCAP or SMART-COP or SWAT-Bp or REA-ICU or clinical prediction rule$ or clinical prediction score$ or clinical prediction tool$ or clinical decision rule$ or clinical decision score$ or severity assessment score$ or Model$ or risk stratification$ or Prognostic scoring system$ or Severity prognosis or Risk score$ or Severity index or Prognostic tool$ or Severity of illness index or clinical decision support system$ or clinical decision system$).ti,ab.	6,159,592
15	(Developing OR less$ developed OR under developed OR underdeveloped OR underserved OR under served OR deprived OR poor$ OR least economically developed OR nonindustriali?ed OR non-industriali?ed OR middle income OR low$ income OR lower middle-income OR low-middle-income OR Low middle income OR global south OR third world OR resource$ poor OR resource$ limited OR scarce$ resource$ OR low resourced OR LAMI) adj (countr$ OR nation$ OR population$ OR world OR setting$).ti,ab.	136,715
16	(LAMIC$ OR LMIC$ OR Poverty area$ OR Malawi OR Uganda OR Kenya OR Sub-Saharan OR Africa OR Asia).ti,ab.	232,676
17	(low adj3 middle adj3 countr$).ti,ab.	18,550
18	(low$ adj (gdp or gnp or gross domestic or gross national)).ti,ab.	348
19	Transitional countr$.ti,ab.	228
20	1 OR 2 OR 3 OR 13	383,925
21	4 OR 5 OR 6 OR 7 OR 8 OR 14	7,445,811
22	9 OR 10 OR 11 OR 12 OR 15 OR 16 OR 17 OR 18 OR 19	475,026
23	20 AND 21 AND 22	3,209
24	Limit 23 to English language	3,171

Cochrane Central Register of Controlled Trials: May 21, 2020

#	Search terms	Results
1	Pneumonia	15,516
2	Community-Acquired Pneumonia	1,585
3	Respiratory Tract Infections	5,850
4	Risk Assessment	62,215
5	"Severity of Illness Index"	18,897
6	Prognosis	32,906
7	Decision Making	15,785
8	Decision Trees	2,147
9	Developing Countries	6,302
10	Poverty Areas	678
11	Poverty	2,828
12	Africa	9,963
13	"Africa South of the Sahara"	302
14	Sub-Saharan	1,898
15	Asia	7,626
16	(Pneumonia OR Community-acquired pneumonia OR CAP OR Community-acquired infection* OR Respiratory tract disease* OR Respiratory infection*).ti,ab.	3,908
17	(Severity score* OR Predict* OR Prognosis OR Mortality score* OR CURB65 OR CURB 65 OR CURB-65 OR C-U-R-B-65 OR CRB65 OR CRB 65 OR CRB-65 OR C-R-B-65 OR Pneumonia severity index OR PSI OR PORT score OR Fine Score OR Tool* OR decision tree OR SOAR OR A-DROP OR PIRO OR RISC OR mRISC OR APPIS OR SCAP OR SMART-COP OR SWAT-Bp OR REA-ICU OR clinical prediction rule* OR clinical prediction score* OR clinical decision rule* OR clinical decision score* OR severity assessment score* OR model* OR risk stratification* OR Prognostic scoring system* OR Severity prognosis OR Risk score* OR Severity index OR Prognostic tool* OR Severity of illness index OR clinical decision support system* OR clinical decision system*).ti,ab.	3,908
18	(Developing OR less* developed OR under developed OR underdeveloped OR underserved OR under served OR deprived OR poor* OR least economically developed OR nonindustriali?ed OR non-industriali?ed OR middle income OR low* income OR lower middle-income OR low-middle-income OR Low middle income OR global south OR third world OR resource* poor OR resource* limited OR scarce* resource* OR low resourced OR LAMI) adj (countr* OR nation* OR population* OR world OR setting*).ti,ab.	4,980
19	(LAMIC* OR LMIC* OR Poverty area* OR Malawi OR Uganda OR Kenya OR Sub-Saharan OR Africa OR Asia).ti,ab.	3,908
20	(low adj3 middle adj3 countr*).ti,ab.	3,908
21	(low* adj (gdp or gnp or gross domestic or gross national)).ti,ab.	3,908
22	MeSH descriptor: [Pneumonia] explode all trees	3504
23	MeSH descriptor: [Risk Assessment] explode all trees	8616
24	MeSH descriptor: [Clinical Decision-Making] explode all trees	198
25	MeSH descriptor: [Prognosis] explode all trees	149253
26	MeSH descriptor: [Developing Countries] explode all trees	830
27	MeSH descriptor: [Poverty] explode all trees	1662
28	MeSH descriptor: [Africa South of the Sahara] explode all trees	6068
29	MeSH descriptor: [Africa] explode all trees	6700
30	MeSH descriptor: [Asia] explode all trees	18792
31	#1 OR #2 OR #3 OR 16 OR #22	225215
32	#4 OR #5 OR #6 OR #7 OR #8 OR #17 OR #23 OR #24 OR #25	234188
33	#9 OR #10 OR #11 OR #12 OR #13 OR #14 OR #15 OR #18 OR #19 OR #20 OR #21 OR #26 OR #27 OR #28 OR #29 OR #30	48185
34	#31 AND #32 AND #33 in Trials	2805

Table S1. Additional study characteristics

Authors	Enrolment period	Inclusion criteria	Exclusion criteria	CAP definition
Kabundji et al., 2014	Between February 2011 and April 2011	Adult patients aged ≥18 years with CAP	• Suspected or confirmed aspiration pneumonia, chemical pneumonitis, *Pneumocystis jirovecii* pneumonia and pulmonary tuberculosis.<br>• Patients with any acute or active comorbid illness such as diabetes mellitus, renal failure, cardiac failure or end-stage AIDS	Two or more of the following: altered breath sounds +/- signs of lung consolidation, fever, rigours, sweats and cough, with or without sputum production, pleuritic chest pain, cyanosis, shortness of breath and tachypnoea, together with radiological confirmation of the diagnosis of pneumonia
Birkhamshaw et al., 2013	Over two months in February and March 2010	All patients presenting with a clinical diagnosis of acute LRTI with a presumptive diagnosis of CAP	• If LRTI was not the primary diagnosis;<br>• If they had recently been hospitalised within 14 days presentation; or<br>• If the patient had known malignancy	A presumptive diagnosis of CAP was made if; at least two recognised signs or symptoms of pneumonia; with an illness duration of 21 days or less; and LRTI/CAP was the primary diagnosis recorded by the attending clinician
Millman et al., 2017	January 2010 through December 2011	Patients ≥18 years of age hospitalised with LRTI	-	SARI Definition: acute LRTI, defined as a hospitalised case in persons ≥ 5 years of age who met a modified 2011 World Health Organization (WHO) case definition for severe acute respiratory infection: sudden onset of fever (>38 °C) or reported fever, cough or sore throat, and shortness of breath or difficulty breathing with illness onset within seven days before admission
Shah et al., 2010	-	Patients presenting with CAP	• Known HIV positive patients<br>• Chronically immunosuppressed patients (immunosuppression for solid organ transplantation, post-splenectomy, receiving >10mg/day of prednisone or the equivalent for more than 30 days, treatment with other immunosuppressive agents, neutropenia with absolute neutrophil count <1000/mm3)<br>• Hospitalised within the previous 14 days<br>• Patients with an alternate diagnosis during follow-up	Patients presenting with any opacity on chest radiograph consistent with the diagnosis of acute pneumonia, associated with respiratory symptoms, infectious syndrome, and lack of an alternate diagnosis
Zuberi et al., 2008	Between October 2006 and May 2007	Patients aged ≥16 years who required admission to the Emergency Room (ER) for the management of CAP	• If pneumonia was not the primary cause of admission<br>• Patients had post-obstructive pneumonia<br>• Tuberculosis<br>• Bronchiectasis<br>• Solid organ and haematological malignancies or HIV infection<br>• Immunocompromised patients and nursing home residents	An acute lung parenchymal infection with clinical and chest radiographic evidence on admission consistent with an infection that was not pre-existing or of any other known cause in a patient not hospitalised for >14 days before the onset of symptoms
Mbata et al., 2014	From December 2008 to June 2009	Adults aged >18 years if they have two or more symptoms and infiltrates on the chest x-ray	• Patients admitted to the hospital in the previous 14 days<br>• Those whose symptoms developed 48 hrs after admission<br>• Patients with tuberculosis or a previous chest x-ray which may conflict with a diagnosis of CAP<br>• Patients who were unwilling to participate	An acute infection of the pulmonary parenchyma associated with symptoms and signs of acute infection, followed by the presence of an acute infiltrate on the chest x-ray in a patient who was not resident in a hospital or healthcare facility in the previous 14 days

Table S1. Additional study characteristics | continued

Authors	Enrolment period	Inclusion criteria	Exclusion criteria	CAP definition
Buss et al., 2018	Between 19th February and 1st April or 6th October and 27th November 2011	Adults ≥16 years admitted with a preliminary clinical diagnosis of CAP (a clinical presentation of LRTI and a presumed primary diagnosis of CAP on admission)	• Alternative primary admission diagnosis (not CAP)<br>• Hospitalisation within the preceding 14 days (excluding hospital-acquired pneumonia)<br>• Pneumonia as an expected terminal event in disseminated Kaposi's sarcoma<br>• Symptoms for more than 21 days	Following the British Thoracic Society primary care guidelines:<br>• Symptoms of acute lower respiratory tract illness<br>• New focal chest signs on examination<br>• Evidence of systemic illness<br>• No other explanation for the illness<br>• A clinical decision that it should be treated with antibiotics
Koss et al., 2015	September 2008 to March 2011	Adults at least 18 years of age with suspected HIV, cough for at least two weeks but fewer than six months, and clinically suspected pneumonia	• Patients with a reduced level of consciousness<br>• Patients who were already receiving treatment for TB or who tested negative for HIV infection	-
Abd-El-Gawad et al., 2013	From October 2011 to June 2012	Patients aged ≥60 presenting with CAP and admitted consecutively to geriatric and chest ICUs	• Hospital-, nursing home-, or ventilator-acquired pneumonia<br>• Severe immunosuppression<br>• Hospitalised within the previous four weeks<br>• Had antibiotics within the previous two weeks<br>• There were any missing data.	Based on the Institute for Clinical Systems Improvement Health Care Guideline: patients with two or more of rigours, pleuritic chest pain, shortness of breath, chest tightness, deep cough, sputum production, fever >37.8°C lasting >72 hours, night sweats, and wheezing. Besides, temperature >37.8°C, pulse >100 beats/minute, decreased breath sounds, rales, or respiratory rate >20 breath/minute were considered. Chest X-ray (CXR) was obtained, and pneumonia was confirmed if showed lung infiltration
Aston et al., 2019	Between May 15, 2013, and January 31, 2015	Adults (>18 years) hospitalised with clinically diagnosed CAP	• Symptoms for >14 days<br>• Suspected co-existent meningitis<br>• Pre-admission diagnosis of terminal illness (e.g., metastatic malignancy, terminal AIDS)<br>• Current anti-tuberculous treatment<br>• Admission to hospital >24 hours previously<br>• Prior hospitalisation within the preceding four weeks<br>• Prior participation in the study	Reported or recorded fever (≥38°C), at least one relevant symptom (cough, chest pain, breathlessness, haemoptysis), and at least one focal chest sign (crepitations, pleural rub, bronchial breathing, percussive dullness, or diminished breath sounds)
Rajarajan et al., 2017	-	Patients presenting with CAP	• Patients < 12 years<br>• Pregnant women<br>• HIV patients<br>• Sputum positive pulmonary TB patients<br>• Chronically immunosuppressed patients (solid organ transplanted, post-splenectomy)<br>• Hospitalised within the previous 14 days for other illness<br>• Patients with an alternate diagnosis during follow-up	Any opacity on chest radiograph associated with respiratory symptoms and signs suggestive of pneumonia

Table S2. Quality assessment of included studies

Study	Study participation	Study attrition	Prognostic factor measurement	Outcome measurement	Study confounding	Statistical analysis and reporting	Overall	Overall quality
Kabundji et al., 2014	1	2	2	2	0	0	7	Poor
Birkhamshaw et al., 2013	2	1	2	2	2	2	11	Good
Millman et al., 2017	2	2	2	2	2	2	12	Good
Shah et al., 2010	1	2	2	2	1	2	10	Moderate
Zuberi et al., 2008	2	1	2	2	2	2	11	Good
Mbata et al., 2014	1	2	2	2	2	2	11	Good
Buss et al., 2018	2	1	2	2	0	2	9	Moderate
Koss et al., 2015	2	1	2	2	2	1	10	Moderate
Abd-El-Gawad et al., 2013	1	2	1	2	1	2	9	Moderate
Aston et al., 2019	2	1	2	2	2	2	11	Good
Rajarajan et al., 2017	0	2	2	2	0	0	6	Poor

Where studies were considered of:

- Good quality or low risk of bias if overall score 11-12
- Moderate quality or moderate risk of bias if overall score 9-10
- Poor quality or high risk of bias if overall score ≤8

Table S3. Components of identified scoring systems

Score	Components
CURB-65	One point for each of the following:<br>• Confusion<br>• Urea >7 mmol/l<br>• Respiratory rate of ≥30 breaths/min<br>• Systolic blood pressure <90 mmHg or diastolic blood pressure ≤60 mmHg<br>• Age ≥65<br>o 0 or 1: low risk (less than 3% mortality risk)<br>o 2: intermediate risk (3% to 15% mortality risk)<br>o 3 to 5: high risk (more than 15% mortality risk)
CRB-65	Used in primary care – (widely used in Europe for hospitalised patients).<br>One point for each of the following:<br>• Confusion<br>• Respiratory rate of ≥30 breaths/min<br>• Systolic blood pressure <90 mmHg or diastolic blood pressure ≤60 mmHg<br>• Age ≥65<br>o 0: low risk (less than 1% mortality risk)<br>o 1 or 2: intermediate risk (1% to 10% mortality risk)<br>o 3 or 4: high risk (more than 10% mortality risk)
PSI	A 20-point score classifies patients into five risk categories (I-V).<br>• Age – (1 point/year, – 10 if female)<br>• Nursing home resident – (10 points)<br>• Neoplastic disease – (30 points)<br>• Liver disease – (20 points)<br>• Congestive heart failure – (10 points)<br>• Cerebrovascular disease – (10 points)<br>• Renal disease – (10 points)<br>• Altered mental status – (20 points)<br>• Pulse ≥125/min – (10 points)<br>• Respiratory rate >30 breaths/min – (20 points)<br>• Systolic blood pressure <90 mmHg – (20 points)<br>• Temperature <35 or ≥40°C – (15 points)<br>• Arterial pH <7.35 – (30 points)<br>• Urea ≥30 mg/dl – (20 points)<br>• Sodium <130 mmol/L – (20 points)<br>• Glucose ≥250 mg/dl – (10 points)<br>• Haematocrit <30% – (10 points)<br>• PaO_2 ≥60 mmHg – (10 points)<br>• Pleural effusion – (10 points)<br>o Class I – (<51 points) – Outpatient treatment<br>o Class II – (51 - 70 points) – Outpatient treatment<br>o Class III – (71 - 90 points) – Outpatient or inpatient treatment<br>o Class IV – (91 - 130 points) – Inpatient treatment<br>o Class V – (131 - 395 points) – Inpatient treatment
SWAT-Bp	• Male sex – (1 point)<br>• Muscle wasting – (1 point)<br>• Non-ambulatory – (1 point)<br>• Temperature (>38°C or <35°C) – (1 point)<br>• Blood pressure (systolic <100 and/or diastolic <60) – (1 point)<br>o 0-1point: home or early discharge<br>o 2 points: hospital treatment<br>o 3-5 points: urgent hospitalization and treatment
SMRT-CO	• Systolic blood pressure < 90 mmHg – (2 points)<br>• Multilobar chest x-ray involvement – (1 point)<br>• Respiratory rate raised (≥25 breaths/min if age ≤50 years and ≥30 breaths/min if age >50 years) – (1 point)<br>• Tachycardia > 125 beats/min – (1 point)<br>• Confusion (new onset) – (1 point)<br>• Oxygen low (<70 mmHg if age ≤50 years or <60 mmHg if age >50 years) – (2 points)
Modified IDSA/ATS minor criteria	• Respiratory rate ≥30/min<br>• Oxygen saturations ≤90% (used as a surrogate for PaO_2/FIO_2 ratio ≤250 criterion)<br>• Multilobar infiltrates<br>• Confusion / disorientation<br>• Urea ≥7.1 mmol/L<br>• White blood cell count < $4x10^9$ cells/L<br>• Platelets <100 10^9 cells/L<br>• Temperature <36° C<br>• Systolic blood pressure <90 mmHg (a surrogate for hypotension requiring aggressive fluid resuscitation)

Table S3. Components of identified scoring systems | continued

Score	Components
CURB-45	One point for each of the following:<br>• Confusion<br>• Urea >7 mmol/l<br>• Respiratory rate of ≥30 breaths/min<br>• Systolic blood pressure <90 mmHg or diastolic blood pressure ≤60 mmHg<br>• Age ≥45
ADL score	A score range from 0 to 6<br>• Dependent feeding<br>• Dependent bathing<br>• Dependent dressing<br>• Dependent continence<br>• Dependent transfer<br>• Dependent toileting
SCAP	Defines severe CAP if one or more major criteria or two or more minor criteria.<br>Major criteria:<br>• Arterial pH <7.30<br>• Systolic blood pressure <90 mmHg<br>Minor criteria:<br>• Respiratory rate >30 breaths/min<br>• Blood urea nitrogen >30 mg/dL<br>• Confusion<br>• Oxygen arterial pressure <54 mmHg or PaO_2/FiO_2 <250mmHg<br>• Age ≥80 years<br>• Multilobar/bilateral lung affectation
Koss et al.	A 4-variable predictor score (1 point per clinical feature, range from 0 – 4)<br>• Heart rate >120 beats/minute<br>• Respiratory rate >30 breaths/ minute<br>• Oxygen saturation <90%<br>• CD4 cell count <50 cells/mm<br>○ 0 or 1: low mortality<br>○ 2 or 3: intermediate mortality<br>○ 4: high mortality
CTA	• Neurologic symptoms<br>• Respiratory rate ≥ 25/min<br>• Creatinine >1.2 mg/dl<br>○ Stage 1 (No symptoms) – 1 score<br>○ Stage 2 (Respiratory rate) – 2 scores<br>○ Stage 3 (Neurologic symptoms) – 3 scores<br>○ Stage 4 (Respiratory rate and creatinine) – 4 scores<br>○ Stage 5 (Neurologic symptoms and respiratory rate) – 5 scores
ACHU score	A 7-point ACHU clinical predictor score range from 0 to 6<br>• Age≥45 (1 point)<br>• Confusion (2 points)<br>• HIV-infected (1 point)<br>• Urea > 7 mmol/L (2 point)<br>○ Low ≤1<br>○ Low-intermediate 2<br>○ High-intermediate 3<br>○ High ≥4

Table S4. Summary of the extracted results

Study ID	Assessed outcome	Assessed score	Sample size	Findings
Kabundji et al., 2014	• Need for admission<br>• Time to clinical stability<br>• Mortality	• CRB-65	152	Outpatient: 84 (55.3%), inpatient: 68 (44.7%)<br>Low-risk patients: 107<br>Died: 0<br>Time to clinical stability:<br>• 1 day: 16 (47%)<br>• 2 days: 17 (50%)<br>• 3 days: 1 (3%)<br>Intermediate risk patients: 42<br>Died: 3 (7%)<br>Time to clinical stability:<br>• 1 day: 6 (21.5%)<br>• 2 days: 16 (57.0%)<br>• 3 days: 6 (21.5%)<br>High risk patients: 3<br>Died: 2 (67%)<br>Time to clinical stability:<br>• 1 day: -<br>• 2 days: 1 (100%)<br>• 3 days: -
Birkhamshaw et al., 2013	• Mortality	• CRB-54<br>• SWAT-Bp	240	CRB-65<br>AUROC: 0.649<br>≥0: Sensitivity: 100%, Specificity: -, PPV: 18.3%, NPV: 2%<br>≥1: Sensitivity: 90.1%, Specificity: 30.6%, PPV: 22.7%, NPV: 93.8%<br>≥2: Sensitivity: 36.4%, Specificity: 80.6%, PPV: 29.6%, NPV: 84.9%<br>≥3: Sensitivity: 6.8%, Specificity: 98%, PPV: 42.9%, NPV: 82.4%<br>≥4: Sensitivity: 2.3%, Specificity: 100%, PPV: 100%, NPV: 8%<br>SWAT-Bp<br>AUROC: 0.867<br>≥0: Sensitivity: 100%, Specificity: -, PPV: 18.3%, NPV: -<br>≥1: Sensitivity: 100%, Specificity: 14.8%, PPV: 20.9%, NPV: 100%<br>≥2: Sensitivity: 95.5%, Specificity: 44.9%, PPV: 28%, NPV: 97.8%<br>≥3: Sensitivity: 84.1%, Specificity: 77%, PPV: 45.1%, NPV: 95.8%<br>≥4: Sensitivity: 52.3%, Specificity: 94.4%, PPV: 67.6%, NPV: 90.7%<br>5: Sensitivity: 1%, Specificity: 99.5%, PPV: 87.5%, NPV: 86.2%
Millman et al., 2017	• In-hospital mortality	• CURB-65: 1011<br>• CRB-65: 1332<br>• CTA: 1011<br>• CURB-45: 1011<br>• ACHU: 1011	1356 randomly selected SARI patients	CURB-65: c-statistic: 0.594<br>CRB-65: c-statistic: 0.548<br>CTA: c-statistic: 0.569<br>CURB-45: c-statistic: 0.666<br>ACHU: c-statistic: 0.769<br>Final model discrimination c-statistic: 0.789<br>Calibration (chi-square 1.6, Hosmer-Lemeshow goodness-of-fit p-value = 0.904)
Koss et al., 2015	• 30-day mortality	• New score	853	For each point, mortality increases by 65% (OR 1.65, 95% CI 1.39-1.96, p <0.001)<br>Calibration $\chi2$ = 1.12 (p = 0.98)

Table S4. Summary of the extracted results | continued

Study ID	Assessed outcome	Assessed score	Sample size	Findings
Shah et al., 2010	• Mortality<br>• ICU admission (marker for severe pneumonia)	• PSI<br>• CURB-65	150	PSI - Mortality<br>I: Sensitivity: -, Specificity: -, PPV: -, NPV: -<br>II: Sensitivity: 100%, Specificity: 18.7%, PPV: 12.8%, NPV: 100%<br>III: Sensitivity: 100%, Specificity: 38.8%, PPV: 16.3%, NPV: 100%<br>IV: Sensitivity: 100%, Specificity: 52.2%, PPV: 20%, NPV: 100%<br>V: Sensitivity: 50%, Specificity: 88.8%, PPV: 34.8%, NPV: 93.7%<br>PSI - ICU admission<br>I: Sensitivity: -, Specificity: -, PPV: -, NPV: -<br>II: Sensitivity: 100%, Specificity: 21.7%, PPV: 28%, NPV: 100%<br>III: Sensitivity: 100%, Specificity: 45.2%, PPV: 35.7%, NPV: 100%<br>IV: Sensitivity: 100%, Specificity: 60.9%, PPV: 43.8%, NPV: 100%<br>V: Sensitivity: 48.6%, Specificity: 94.8%, PPV: 73.9%, NPV: 85.8%<br>CURB-65 - Mortality<br>0: Sensitivity: -, Specificity: -, PPV: -, NPV: -<br>1: • Sensitivity: 100%, Specificity: 20.1%, PPV: 13%, NPV: 100%<br>2: • Sensitivity: 100%, Specificity: 43.3%, PPV: 17.4%, NPV: 100%<br>3: • Sensitivity: 100%, Specificity: 74.6%, PPV: 32%, NPV: 100%<br>4: • Sensitivity: 87.5%, Specificity: 88.8%, PPV: 48.3%, NPV: 98.3%<br>5: • Sensitivity: 18.8%, Specificity: 97.8%, PPV: 50%, NPV: 91%<br>CURB-65 - ICU admission<br>0: Sensitivity: -, Specificity: -, PPV: -, NPV: -<br>1: Sensitivity: 100%, Specificity: 23.5%, PPV: 28.5%, NPV: 100%<br>2: Sensitivity: 100%, Specificity: 50.4%, PPV: 38%, NPV: 100%<br>3: Sensitivity: 91.4%, Specificity: 84.4%, PPV: 64%, NPV: 97%<br>4: Sensitivity: 74.3%, Specificity: 97.4%, PPV: 89.7%, NPV: 92.6%<br>5: Sensitivity: 17.1%, Specificity: 100%, PPV: 100%, NPV: 79.9%
Zuberi et al., 2008	• Mortality	• CURB-65<br>• CRB-65	137	CURB-65 - AUROC: 0.863<br>≥0: Sensitivity: 100%, Specificity: 0, PPV: 12.7%, NPV: NC<br>≥1: Sensitivity: 100%, Specificity: 22%, PPV: 16%, NPV: NC<br>≥2: Sensitivity: 100%, Specificity: 53%, PPV: 24%, NPV: 100%<br>≥3: Sensitivity: 78%, Specificity: 82%, PPV: 38%, NPV: 96%<br>≥4: Sensitivity: 17%, Specificity: 97%, PPV: 42%, NPV: 88%<br>5: Sensitivity: 6%, Specificity: 100%, PPV: 100%, NPV: 87%<br>OR (95% CI) for high vs combined low and intermediate risk (reference): 15.4 (4.6-51.4)<br>CRB-65 - AUROC: 0.835<br>≥0: Sensitivity: 100%, Specificity: 0, PPV: 13%, NPV: NC<br>≥1: Sensitivity: 100%, Specificity: 29%, PPV: 17%, NPV: NC<br>≥2: Sensitivity: 83%, Specificity: 72%, PPV: 31%, NPV: 96%<br>≥3: Sensitivity: 28%, Specificity: 97%, PPV: 55%, NPV: 89%<br>4: Sensitivity: 6%, Specificity: 100%, PPV: 100%, NPV: 87%<br>OR (95% CI) for high vs combined low and intermediate risk (reference): 11.1 (2.6-46.4)
Rajarajan et al., 2017	• Mortality	• PSI	50	Only one patient died during the hospital stay, and he was classified as PSI Class V (Severe)

Table S4. Summary of the extracted results | continued

Study ID	Assessed outcome	Assessed score	Sample size	Findings
Mbata et al., 2014	• Mortality<br>• The need for ICU admission	• CURB-65<br>• CRB-65	80	<u>CURB-65, mortality</u><br>0: Sensitivity: 100%, Specificity: 19.1%, PPV: 17.9%, NPV: 100%<br>1: Sensitivity: 91.7%, Specificity: 45.6%, PPV: 22.9%, NPV: 96.9%<br>2: Sensitivity: 83.3%, Specificity: 23.5%, PPV: 16.1%, NPV: 88.9%<br>3: Sensitivity: 58.3%, Specificity: 20.6%, PPV: 11.5%, NPV: 73.7%<br>4: Sensitivity: 66.7%, Specificity: 9.7%, PPV: 11.4%, NPV: 60%<br>5: Sensitivity: NA, Specificity: NA, PPV: NA, NPV: NA<br><u>CRB-65, mortality</u><br>0: Sensitivity: 100%, Specificity: 19.1%, PPV: 17.9%, NPV: 100%<br>1: Sensitivity: 91.7%, Specificity: 51.5%, PPV: 25%, NPV: 97.2%<br>2: Sensitivity: 58.3%, Specificity: 33.8%, PPV: 13.5%, NPV: 82.1%<br>3: Sensitivity: 58.3%, Specificity: 13.3%, PPV: 10.4%, NPV: 61.5%<br>4: Sensitivity: 91.7%, Specificity: 1.5%, PPV: 14.1%, NPV: 50%
Buss et al., 2018	• Mortality	• SWAT-Bp	216	• AUROC: 0.744<br>≥0: Sensitivity: 100% (84.5-100) Specificity: 0 (0-2.5), PPV: 12.5% (8.5-17.8), NPP: -<br>≥1: Sensitivity: 100% (84.5-100), Specificity: 22.8% (17.1-30), PPV: 15.6% (10.7-22.1), NPV: 100% (89.8-100)<br>≥2: Sensitivity: 81.5% (61.3-93.0), Specificity: 51.3% (44-58.6), PPV: 19.3% (12.7-28.0), NPV: 95.1% (88.4-98.2)<br>≥3: Sensitivity: 55.6% (35.6-74.0), Specificity: 76.7% (70-82.4), PPV: 25.4% (15.4-38.7), NPV: 92.4% (86.7-95.8)<br>≥4: Sensitivity: 25.9% (11.9-46.6), Specificity: 94.7% (90.2-97.3), PPV: 41.2% (19.4-66.5), NPV: 89.9% (84.7-93.6)<br>5: Sensitivity: 11.1% (2.9-30.3), Specificity: 100% (97.5-100), PPV: 100% (31-100), NPV: 88.7% (83.5-92.5)
Abd-El-Gawad et al., 2013	• 30-day mortality<br>• Mechanical ventilation (MV)	• ADL score ≤3<br>• CURB-65≥3<br>• Positive SCAP	65	<u>ADL</u><br>30-day mortality: Sensitivity: 100%, Specificity: 41%, PPV: 53%, NPV: 100%, AUROC: 0.705<br>MV: Sensitivity: 96.3%, Specificity: 39.5%, PPV: 53.1%, NPV: 93.8%, AUROC: 0.679<br><u>CURB-65</u><br>30-day mortality: Sensitivity: 50%, Specificity: 87.2%, PPV: 72.2%, NPV: 72.3%, AUROC: 0.686<br>MV: Sensitivity: 44.44%, Specificity: 84.2%, PPV: 66.67%, NPV: 68.09%, AUROC: 0.643<br><u>SCAP</u><br>30-day mortality: Sensitivity: 65.4%, Specificity: 66.7%, PPV: 56.7%, NPV: 74.3%, AUROC: 0.660<br>MV: Sensitivity: 77.7%, Specificity: 76.3%, PPV: 70%, NPV: 82.86%, AUROC: 0.770
Aston et al., 2019	• 30-day mortality	• CURB65 ≥3: 412<br>• CRB65 ≥2: 421<br>• SMRT-CO ≥2: 280<br>• Modified IDSA/ATS minor criteria ≥3: 272<br>• SWAT-Bp ≥3: 427	459	<u>CURB-65</u><br>Sensitivity: 14% (6.3–25.8), Specificity: 91.5% (88.2–94.2), PPV: 21.1% (9.6–37.3), NPV: 86.9% (83.1–90.1), PLR: 1.66 (0.80–3.44), NLR: 0.94 (0.84–1.05), AUROC: 0.6 (0.52–0.67)<br><u>CRB-65</u><br>Sensitivity: 27.9% (17.1–40.8), Specificity: 81.9% (77.6–85.8), PPV: 20.7% (12.6–31.1), NPV: 87% (83.0–90.4), PLR: 1.54 (0.97–2.44), NLR: 0.88 (0.75–1.04), AUROC: 0.57 (0.50–0.65)<br><u>SMRT-CO</u><br>Sensitivity: 89.7% (72.6–97.8), Specificity: 36.7% (30.7–42.9), PPV: 14.1% (9.4–19.9), NPV: 96.8% (91.0–99.3), PLR: 1.42 (1.21–1.65), NLR: 0.28 (0.10–0.83), AUROC: 0.66 (0.57–0.75)<br><u>Modified IDSA/ATS minor criteria</u><br>• Sensitivity: 48.3% (29.4–67.5), Specificity: 72% (65.9–77.6), PPV: 17.1% (9.7–27.0), NPV: 92.1% (87.3–95.5), PLR: 1.73 (1.13–2.64), NLR: 0.72 (0.50–1.03), AUROC: 0.66 (0.56–0.75)<br><u>SWAT-Bp</u><br>Sensitivity: 54.1% (40.8–66.9), Specificity: 68.3% (63.3–73.0), PPV: 22.1% (15.8–29.7), NPV: 89.9% (85.8–93.2), PLR: 1.71 (1.30–2.25), NLR: 0.67 (0.51–0.89), AUROC: 0.65 (0.57–0.72)

ICU: Intensive care unit; PPV: Positive predictive value; NPV: Negative predictive value; AUROC: Area under the summary receiver operating characteristic curve; CI: Confidence interval; OR: Odds ratio; PLR: Positive likelihood ratio; NLR: Negative likelihood ratio; NC: Not calculable; NA: Not applicable

Review of the other scores' performance at high-risk cut-offs

The well-known PSI was examined in two articles only, which were based in India. The sensitivity and specificity of PSI-IV to predict mortality were 100% and 52.2% compared to 50% and 88.8% for PSI-V, respectively. Its predictive performance was also studied for ICU admission, where the sensitivity and specificity were 100% and 60.9% for PSI-IV and 48.6% and 94.8% for PSI-V, respectively.[30] Another small-scale study assessed PSI in predicting mortality, where only one patient died in PSI-V.[35]

Additional scores were studied for the mortality prediction, with an AUROC of 0.569, 0.666, and 0.789 for CTA, CURB-45, and ACHU score, respectively.[32] A study conducted in Egypt examined the validity of the activities of daily living (ADL) and severe community-acquired pneumonia (SCAP) scores in predicting mortality and the need for mechanical ventilation in ICU elderly patients. For ADL score ≤3, the AUROCs were 0.705 and 0.679, respectively, while for positive SCAP, the AUROCs were 0.799 and 0.770, respectively.[34] The performance of more scores for the mortality prediction was explored in one study, with an AUROC of 0.66, 0.66, and 0.65 for SMRT-CO ≥2, modified IDSA/ATS minor criteria, and SWAT-Bp, respectively.[33] A derivation study and a validation study investigated SWAT-Bp predictive performance for mortality and showed AUROCs of 0.867 and 0.744, respectively.[18 19] Koss et al. developed a novel tool to predict mortality in HIV patients and found that the risk of death increases by 65% for every point (odds ratio 1.65, 95%CI 1.39-1.96, $p<0.001$).[17] Detailed results at different cut-off values are provided in Table 4A.

CURB-65

In the studies excluded from the meta-analysis, CURB-65 ≥3 performance to predict mortality and mechanical ventilation need in ICU elderly patients was assessed in one study and yielded an AUROC of 0.686 and 0.643, respectively.[34] Another study assessed the score's ability to predict mortality with an AUROC of 0.6.[33]

CRB-65

The CRB-65 ≥2 performance to predict mortality yielded an AUROC of 0.57.[33]

*References in the appendix are presented in the main manuscript.

Forest plots of diagnostic odds ratios (DORs)

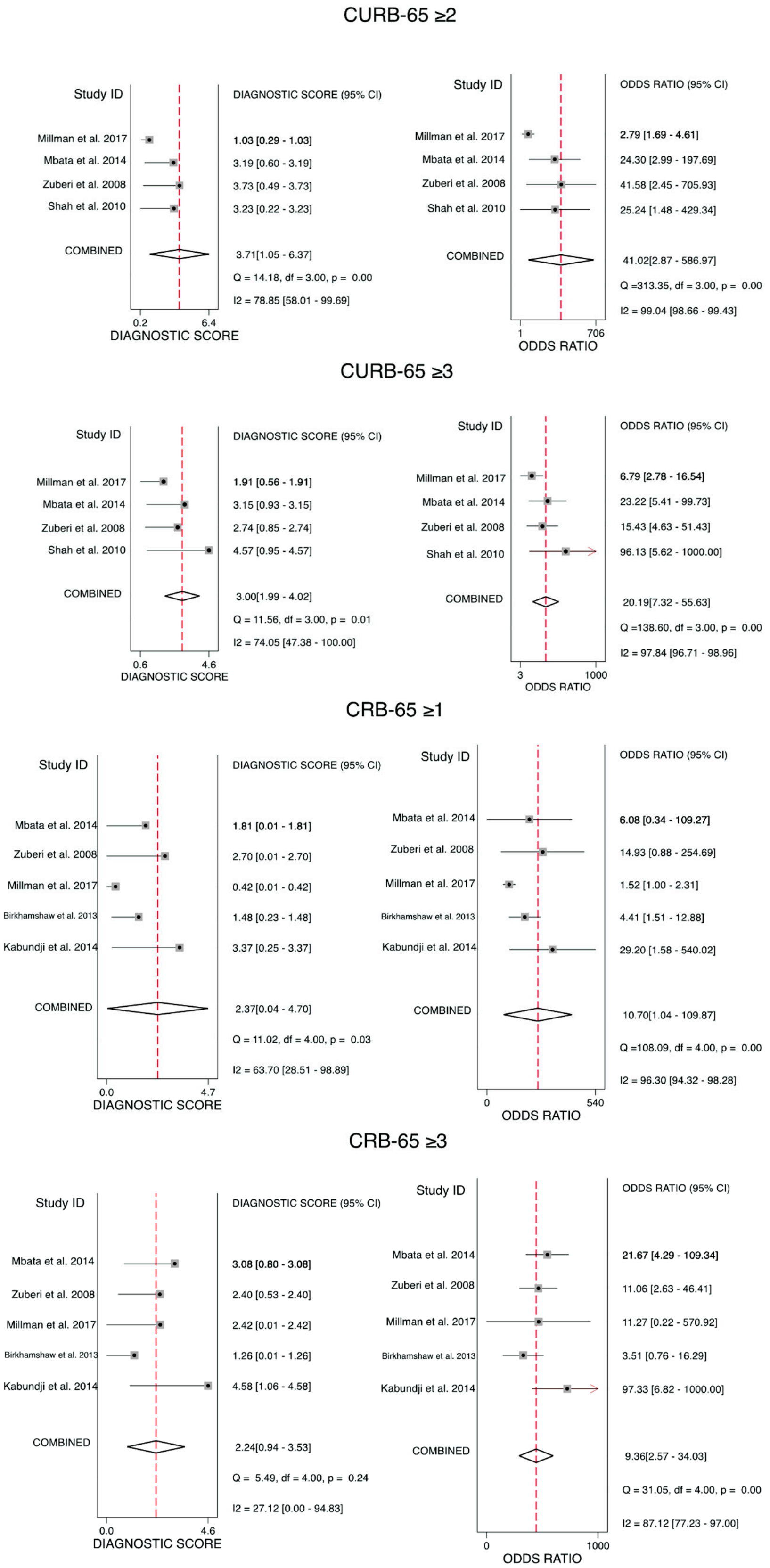

Figure S1. Forest plots for the diagnostic odds ratio (DOR) of CURB-65 and CRB-65 at the studied cut-offs for mortality prediction.

Publication bias

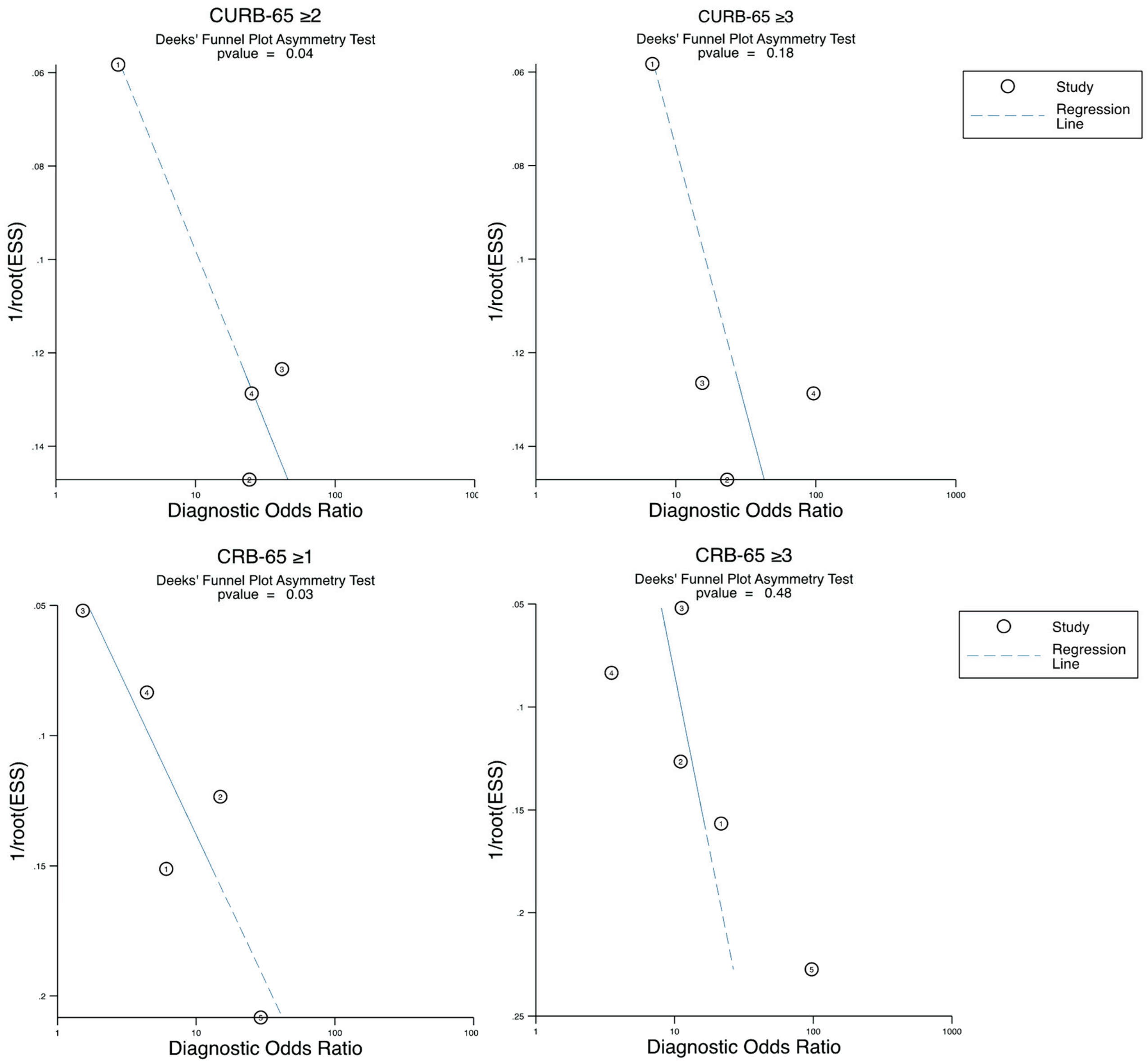

Figure S2. Deek's funnel plots for the assessment of potential publication bias of the included studies.
